# Blockade of Adrenal Medulla-Derived Epinephrine Potentiates Bee Venom-Induced Antinociception in the Mouse Formalin Test: Involvement of Peripheral ****β****-Adrenoceptors

**DOI:** 10.1155/2013/809062

**Published:** 2013-09-08

**Authors:** Suk-Yun Kang, Dae-Hyun Roh, Hyun-Woo Kim, Ho-Jae Han, Alvin J. Beitz, Jang-Hern Lee

**Affiliations:** ^1^Department of Veterinary Physiology, College of Veterinary Medicine and Research Institute for Veterinary Science, Seoul National University, Seoul 151-742, Republic of Korea; ^2^Department of Maxillofacial Tissue Regeneration, School of Dentistry, Kyung Hee University, Seoul 130-701, Republic of Korea; ^3^Department of Physiology and Brain Research Institute, Chungnam National University Medical School, Daejeon 301-747, Republic of Korea; ^4^Department of Veterinary and Biomedical Sciences, College of Veterinary Medicine, University of Minnesota, St. Paul, MN 55108, USA

## Abstract

The injection of diluted bee venom (DBV) into an acupoint has been used traditionally in eastern medicine to treat a variety of inflammatory chronic pain conditions. We have previously shown that DBV had a potent antinociceptive efficacy in several rodent pain models. However, the peripheral mechanisms underlying DBV-induced antinociception remain unclear. The present study was designed to investigate the role of peripheral epinephrine on the DBV-induced antinociceptive effect in the mouse formalin assay. Adrenalectomy significantly enhanced the antinociceptive effect of DBV during the late phase of the formalin test, while chemical sympathectomy had no effect. Intraperitoneal injection of epinephrine blocked this adrenalectomy-induced enhancement of the DBV-induced antinociceptive effect. Moreover, injection of a phenylethanolamine N-methyltransferase (PNMT) inhibitor enhanced the DBV-induced antinociceptive effect. Administration of nonselective **β**-adrenergic antagonists also significantly potentiated this DBV-induced antinociception, in a manner similar to adrenalectomy. These results demonstrate that the antinociceptive effect of DBV treatment can be significantly enhanced by modulation of adrenal medulla-derived epinephrine and this effect is mediated by peripheral **β**-adrenoceptors. Thus, DBV acupoint stimulation in combination with inhibition of peripheral **β**-adrenoceptors could be a potentially novel strategy for the management of inflammatory pain.

## 1. Introduction

In traditional eastern medicine, acupuncture therapy, including manual acupuncture, electroacupuncture, and chemical acupuncture, is commonly used to produce an analgesic effect and to reduce the severity of pain. With regard to chemical acupuncture, it is well established that subcutaneous injection of diluted bee venom (DBV) into an acupoint, termed *apipuncture*, can be used clinically to produce a potent analgesic effect in human patients [1]. Previous experimental studies in our laboratory have provided scientific support for this alternative medicine approach by demonstrating that subcutaneous injection of DBV into the Zusanli (ST36) acupoint produces a robust antinociceptive effect in various animal models of pain including the formalin test [2, 3], the chronic constriction injury model [4, 5], the writhing test [6], and several models of arthritis [7]. We have also demonstrated that acupoint injection of DBV increases Fos expression in brainstem catecholaminergic neurons, including the locus coeruleus [8, 9], and that activation of spinal *α*2-adrenoceptors, but not opioid receptors, is critically involved in this DBV-induced antinociceptive effect [10–12]. Collectively, these results indicate that the antinociceptive effect of DBV treatment is mediated by the activation of descending coeruleospinal noradrenergic pathways and *α*2-adrenoceptors in the spinal cord dorsal horn.

The administration of DBV has also been reported to evoke a significant anti-inflammatory effect in human arthritic disease as well as in several animal models of inflammatory disease [9, 13, 14]. Cumulative studies from our laboratories indicate that the activation of sympathetic preganglionic neurons (SPNs) that innervate the adrenal gland partially underlie DBV's anti-inflammatory action. We also observed that DBV injection into an acupoint activates SPNs leading to release of adrenal medulla-derived epinephrine resulting in a dramatic suppression of neutrophil migration at sites of inflammation [15, 16]. We have also demonstrated that intrathecal injection of clonidine produces an anti-inflammatory effect via the activation of this SPN-adrenomedullary axis, which appears to mimic DBV's anti-inflammatory effect [17]. 

It has been widely accepted that adrenal medulla-derived epinephrine, an endogenous adrenergic receptor ligand, plays a critical role in the maintenance of hyperalgesia, that is, the increased sensitivity to painful stimuli. Khasar et al. reported that an increase in peripheral epinephrine produces cutaneous mechanical hyperalgesia and this nociceptive effect of epinephrine is mediated by both the protein kinase A and protein kinase C second-messenger pathways [18]. Moreover, it is reported that chronically elevated levels of epinephrine led to an enhancement of bradykinin-induced hyperalgesia [19] and that administration of epinephrine to adrenal medullectomized rats reconstituted these enhanced hyperalgesic responses [20]. 

Although we have reported that DBV can produce a significant antinociceptive effect in several pain models, it is not clear whether DBV-induced changes in peripheral epinephrine produce a positive or negative effect on DBV's antinociceptive action. Therefore, this study was designed to investigate the potential role of peripheral epinephrine on the DBV-induced antinociceptive effect in the mouse formalin assay. We first evaluated whether denervation of the adrenal gland or chemical sympathectomy alters the DBV-induced antinociceptive effect in the mouse formalin test using adrenalectomy and 6-hydroxydopamine injection, respectively. We next determined whether epinephrine itself might have an effect on DBV-induced antinociceptive effects in adrenalectomized animals. Finally, we investigated whether this DBV-induced antinociceptive effect could be potentiated by suppression of adrenal medulla-derived epinephrine using the following approaches: (1) inhibition of the PNMT enzyme or (2) antagonism of peripheral epinephrine receptors (*α*- and *β*-adrenoceptors).

## 2. Materials and Methods

### 2.1. Experimental Animals

Experiments were performed on male ICR mice (25 g to 30 g). All experimental animals were obtained from the Laboratory Animal Center of Seoul National University in Republic of Korea. They were housed in colony cages with free access to food and water and maintained in temperature and light controlled rooms (23 ± 20°C, 12/12-hour light/dark cycle with lights on at 7 AM) for at least 1 week before the study. All of the methods used in the present study were approved by the Animal Care and Use Committee at Seoul National University and conform to NIH guidelines (NIH publication No. 86-23, revised 1985). All algesiometric assays were conducted under the ethical guidelines set forth by the International Association for the study of Pain (IASP).

### 2.2. Apipuncture with BV

DBV from *Apis mellifera *(Sigma) was dissolved in physiological saline. In the first experiment, we tested the following three doses of DBV: 0.8 mg/kg (1 K, diluted by saline with ratio of 1 : 1000); 0.08 mg/kg (10 K, diluted by saline with ratio of 1 : 10,000); and 0.008 mg/kg (100 K, diluted by saline with ratio of 1 : 100,000). In subsequent experiments, we used only the 0.8 mg/kg dose of DBV, since this dose produced a significant antinociceptive effect, while the other two doses did not. Each dose of DBV was subcutaneously injected into the left Zusanli acupoint (ST-36) located on the lateral side of the stifle joint adjacent to the anterior tubercle of the tibia as previously described [3]. Animals in the control group received an injection of vehicle into the same site. In all apipuncture experiments, DBV was injected 10 minutes after each drug injection.

### 2.3. Drug Treatment

6-Hydroxydopamine, epinephrine, phentolamine, nadolol, atenolol (Sigma, St. Louis, MO, USA), DCMB, propranolol, and ICI-118,551 (Tocris, UK) were all dissolved in saline (0.9% NaCl) before use. All drugs were intraperitoneally administered in a volume of 100 *μ*L and were injected 10 minutes before DBV injection (20 minutes before formalin injection).

### 2.4. Mice Adrenalectomy

Anesthesia was induced with a Zoletil-Rumpun mixture in saline, in a ratio of 2 : 1 : 2 total volume of anesthetic drug. To remove the adrenal glands, a dorsal midline incision was made. This incision was shifted to either side to expose the anterior pole of the kidney and the adrenal gland. The adrenal gland was removed by separating the gland from the surrounding tissue with tweezers and then gently pulling the gland through the flank incision; 4–0 chromic gut sutures were used to close the incisions in the muscle walls, and silk was used to close the flank incision. After adrenalectomy, 0.9% saline was given to adrenalectomized mice rather than water in order to maintain mineral balance and all animals were allowed to recover on a heating chamber immediately after surgery. All adrenalectomized mice recovered from surgery for a period of one week prior to further experimental use.

### 2.5. Chemical Sympathectomy

In order to examine the contribution of catecholamines released from postganglionic sympathetic nerve endings on DBV-induced antinociception, chemical sympathectomy was performed as previously described by Kohm and Sanders [21]. Experimental mice received a 200 mg/kg intraperitoneal injection of 6-hydroxydopamine (6-OHDA) in sterile saline containing 0.01% ascorbic acid/saline, while control mice received an injection of vehicle. In previous studies, intraperitoneal injection of 6-OHDA has been shown to deplete norepinephrine in the spleen and lymph nodes. In the present study, we confirmed the depletion of catecholamines in sections of spleen using glyoxylic acid staining on day 6 following the injection of 6-OHDA (data not shown).

### 2.6. Formalin-Induced Nociceptive Behavioral Test

The formalin test is an analgesic behavioral observation assessment method that has 2 phases (an early phase and a late one) of nociceptive behavior representing 2 different types of pain. The early phase seems to be caused predominantly by C-fiber activation due to the peripheral stimulus, while the late phase appears to be dependent on the combination of an inflammatory reaction in the peripheral tissue and functional changes in the dorsal horn of the spinal cord. In the present study, mice were first acclimatized for 30 minutes in an acrylic observation chamber (30 cm in diameter and in height), and then 20 *μ*L of 1% formalin was injected subcutaneously into the plantar surface of the left hind paw with a 30 gauge needle. Following formalin injection, the animals were immediately placed in the test chamber, and nociceptive responses in each animal were recorded using a video camera for a period of 30 minutes. The summation of time (in seconds) spent licking the formalin-injected hind paw during each 5-minute block was measured as an indicator of the nociceptive response. Two experienced investigators, who were blinded to the experimental conditions, measured the formalin-induced behaviors. The duration of the responses during the first 10-minute period represented the early phase, while the duration of responses during the subsequent 20-minute period (from 10 to 30 minutes after injection) represented the late phase of the formalin test. 

### 2.7. Statistical Analysis

All values are expressed as the mean ± SEM. Statistical analysis was performed using Prism 5.0 (Graph Pad Software, San Diego, CA, USA). A one-way ANOVA was used to determine differences across all experiment groups. Post hoc analysis was performed using the Bonferroni's multiple comparison test in order to determine the *P* value among experiment groups. *P* < 0.05 was considered statistically significant.

## 3. Results

### 3.1. Effect of DBV Treatment on Formalin-Induced Nociceptive Behaviors in Normal and Adrenalectomized Mice

**Figure 1:**
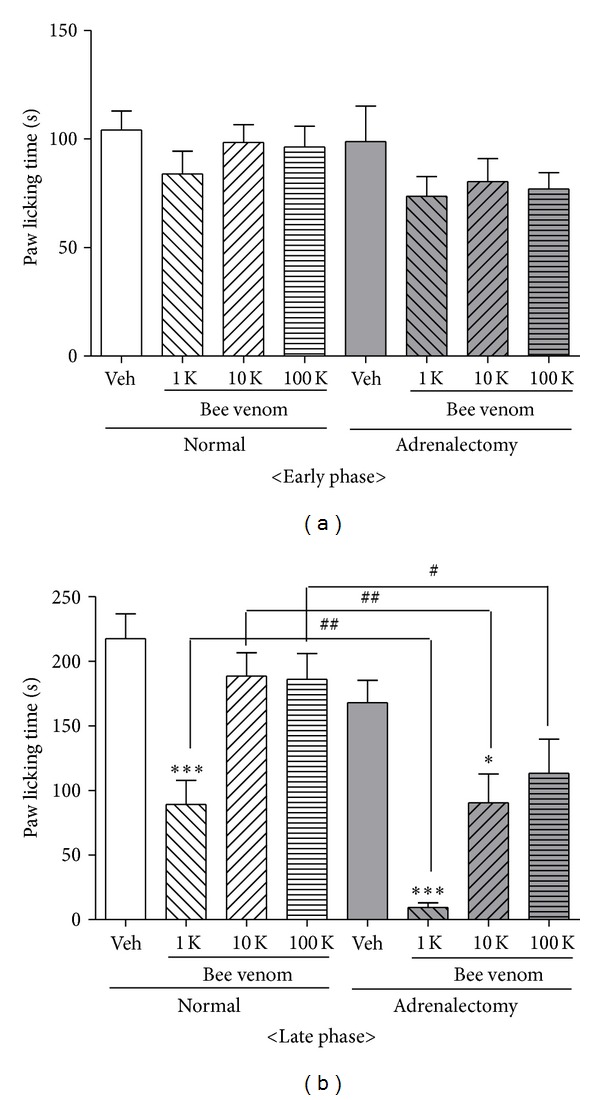
Graphs illustrating the antinociceptive effect of DBV administered at three different doses in normal and adrenalectomized mice, based on analysis of formalin-induced pain responses during the early (a) and late (b) phases of the formalin test. (a) During the early phase, there were no changes in licking behavior in either the normal or adrenalectomized groups treated with DBV at any of the doses tested. (b) During the late phase, administration of a high dose (1 K) of DBV in normal mice significantly inhibited the pain behavior as compared with the vehicle-treated group (***P < 0.001). In adrenalectomized mice, formalin injection produced similar pain responses to those observed in nonadrenalectomized mice. Treatment with DBV (1 K and 10 K) significantly suppressed the paw licking time compared to the vehicle-injected animal group (*P < 0.05 and ***P < 0.001), while the lowest dose (100 K) of DBV had no effect on formalin-induced pain responses. Treatment with each of the three doses (1 K, 10 K, and 100 K) of DBV suppressed pain behavior compared to the same dose of DBV administered to the normal mice (nonadrenalectomized) group (^#^ *P* < 0.05 and ^##^ *P* < 0.01).

We first tested whether subcutaneously injection of DBV into the left Zusanli acupoint produced an antinociceptive effect during the early or late phases of the formalin test using several different doses (1 K, 10 K and 100 K) in normal and adrenalectomized mice. To accomplish this, we first injected DBV and then ten minutes later we injected formalin and subsequently measured licking time of the formalin-treated hind paw. In normal animals, administration of a high dose (1 K, *n* = 7) of DBV significantly decreased formalin-induced paw licking behaviors as compared with the vehicle-injected group (*n* = 7) during the late phase of the formalin test (***P < 0.001, Figure 1(b)). However, DBV had no effect on paw licking time during the early phase of formalin-induced pain behaviors (Figure 1(a)). We also found that administration of lower doses (10 K and 100 K, *n* = 7, resp.) of DBV did not produce any significant antinociceptive effects during either the early or late phase of formalin-induced pain behaviors. In adrenalectomized mice, injection of formalin plus vehicle (rather than DBV) produced similar nociceptive responses to those observed in nonadrenalectomized animals receiving formalin plus vehicle during both the early and late phases of the formalin test. In adrenalectomized mice subcutaneous DBV injection (at 1 K and 10 K, *n* = 7, resp.) significantly reduced the paw licking time compared to that of vehicle-injected animal group (*n* = 7, *P < 0.05 and ***P < 0.001), while the lowest dose (100 K, *n* = 7) of DBV failed to inhibit pain responses during the late phase. However, when compared to the same doses of DBV given to the non-adrenalectomized mice group, administration of DBV to adrenalectomized mice at all doses tested (1 K, 10 K and 100 K) significantly suppressed the paw licking time during the late phase of the formalin test (^#^ *P* < 0.05 and ^##^ *P* < 0.01, Figure 1(b)). Thus, adrenalectomy enhanced the antinociceptive effectiveness of DBV in the late phase of the formalin test. Conversely, DBV-injected into the ST-36 acupoint of adrenalectomized mice did not affect the paw licking time during the early phase of the formalin test.

### 3.2. Lack of Effect of Peripheral Chemical Sympathectomy on DBV-Induced Antinociception

**Figure 2:**
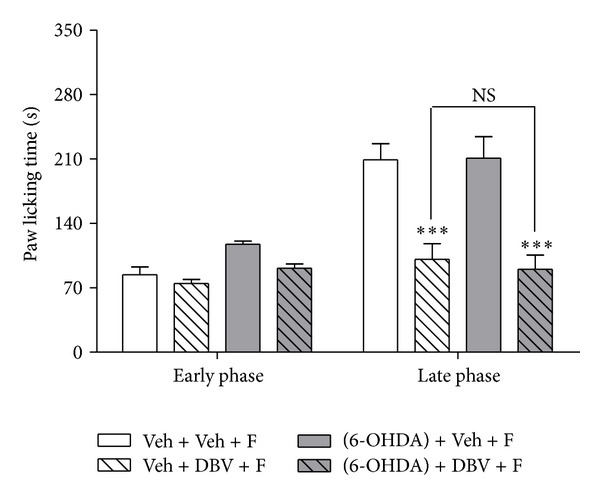
Graphs illustrating the effect of chemical sympathectomy with 6-OHDA in combination with DBV on the formalin test. This figure shows that chemical sympathectomy itself [(6-OHDA) + Veh + F] did not change formalin-induced pain behavior as compared to the vehicle-injected control group (Veh + Veh + F) during either the early or late phase of the formalin test. Injection of 6-OHDA in combination with DBV [(6-OHDA) + DBV + F] also did not affect formalin-induced pain behavior as compared to a DBV-injected control group (Veh + DBV + F, NS; no significance).

We subsequently investigated whether catecholamines released from postganglionic sympathetic nerve endings alter the antinociceptive effects of DBV. To test this we performed a chemical sympathectomy using 6-OHDA. As illustrated in Figure 2, administration of 6-OHDA alone [(6-OHDA) + Veh + F, *n* = 7] did not alter formalin-induced paw licking time as compared to a vehicle-injected control group (Veh + Veh + F, *n* = 7) during either the early or late phases of the formalin test. Similarly, chemical sympathectomy combined with DBV treatment [(6-OHDA) + DBV + F, *n* = 7] did not have any suppressive effect on formalin-induced pain response as compared to a DBV-injected control group (Veh + DBV + F, *n* = 7, NS; no significance). These results indicate that postganglionic sympathetic nerve endings do not play a role in DBV-induced pain reduction during the formalin test.

### 3.3. Effect of Peripheral Epinephrine in DBV-Induced Antinociception

**Figure 3:**
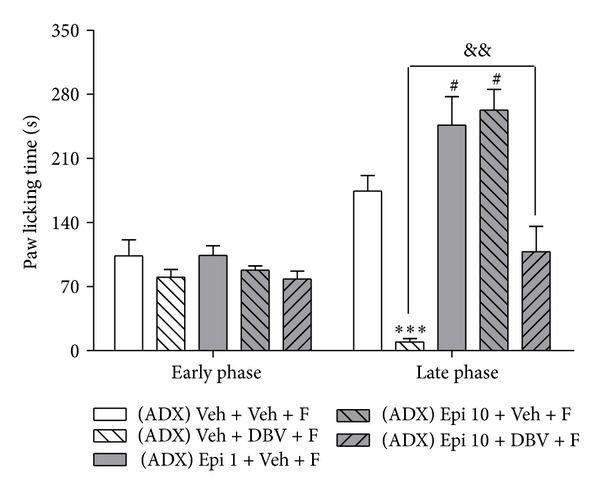
Graph illustrating the effect of administration of exogenous epinephrine in adrenalectomized mice on formalin-induced nociception. Treatment with DBV in adrenalectomized mice (Veh + DBV + F) significantly suppressed pain responses compared to that of the vehicle group (Veh + Veh + F, ***P < 0.001). Epinephrine (1 and 10 ug/kg) was intraperitoneally injected, and this significantly increased formalin-induced pain as compared with the vehicle group during the late phase (^#^ *P* < 0.05). Pretreatment of epinephrine (10 ug/kg) before DBV (Epi 10 + DBV + F) significantly reversed the antinociceptive effect of DBV as compared to the DBV only treated group during the late phase of the formalin test (^&&^ *P* < 0.01).

In order to investigate whether pure epinephrine might have an influence on DBV-induced antinociceptive effects, we performed adrenalectomy and measured the formalin-induced pain behaviors. As illustrated in Figure 3, the DBV-treated group (Veh + DBV + F, *n* = 7) significantly suppressed formalin-induced paw licking behaviors compared to that of vehicle-injected group (Veh + Veh + F, *n* = 7, ***P < 0.001). However, administration of epinephrine (1 and 10 ug/kg, Epi 1 + Veh + F and Epi 10 + Veh + F, *n* = 7, resp.) significantly increased paw licking behavior during the late phase of the formalin test as compared to the vehicle-injected group (^#^ *P* < 0.05). Coadministration of epinephrine (10 ug/kg) with DBV (Epi 10 + DBV + F, *n* = 7) significantly decreased the reduction in paw licking time produced by DBV treatment alone during the late phase of the formalin test (^&&^ *P* < 0.01). Coadministration of epinephrine (10 ug/kg) with DBV (Epi 10 + DBV + F, *n* = 7) had no effect on licking behaviors during the early phase of the formalin test. These results demonstrated that epinephrine might be closely involved in both peripheral sensitization and DBV's antinociception in late phase but not early phase.

### 3.4. Effect of Coadministration of the PNMT Inhibitor: DCMB with DBV on Formalin-Induced Pain Behaviors

**Figure 4:**
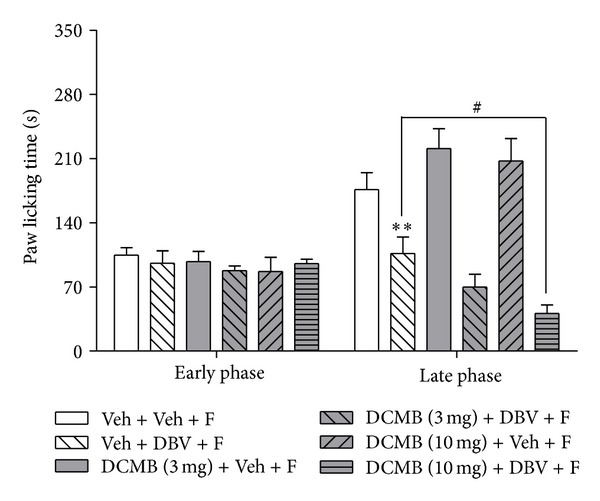
Graphs illustrating the antinociceptive effect of preinjection of the PNMT inhibitor, DCMB, prior to DBV treatment on the formalin test. DBV (Veh + DBV + F) significantly decreased pain behaviors compared to the vehicle group (Veh + Veh + F, **P < 0.01). DCMB-injected groups that were injected with vehicle [DCMB (3, 10 mg) + Veh + F] did not change formalin-induced pain responses during the early and late phases. While a low dose of DCMB given prior to DBV treatment [DCMB (3 mg) + DBV + F] did not change formalin-induced pain responses, a higher dose of DCMB given prior to DBV treatment [DCMB (10 mg) + DBV + F] significantly suppressed formalin-induced pain behaviors during the late phase of the formalin test as compared to that of the DBV-treated group (^#^ *P* < 0.05).

Phenylethanolamine N-methyltransferase (PNMT) is an enzyme that converts norepinephrine to epinephrine in the adrenal medulla. We examined whether administration of the PNMT inhibitor, DCMB, alone or in combination with DBV treatment had any effect on formalin-induced nociceptive responses. Administration of DBV (Veh + DBV + F, *n* = 7) significantly decreased pain behaviors compared to that of vehicle-injected group (Veh + Veh + F, *n* = 7, **P < 0.01). Systemic DCMB injection together with vehicle [DCMB (3, 10 mg) + Veh + F, *n* = 7, resp.] rather than DBV did not change formalin-induced pain behaviors as compared to the vehicle-vehicle-injected group in either the early or late phase of the formalin test (Figure 4). However, when a high dose of DCMB was co-administered with DBV [DCMB (10 mg) + DBV + F, *n* = 7], it significantly increased the antinociceptive effect of DBV on formalin-induced nociceptive responses as compared with the vehicle-DBV-injected group during the late phase of the formalin test (^#^ *P* < 0.05). This drug combination had no effect on the early phase of the formalin test. Moreover, administration of a low dose of DCMB given prior to DBV treatment [DCMB (3 mg) + DBV + F, *n* = 7] did not change formalin-induced pain responses.

### 3.5. Effect of Coadministration of Adrenoceptor Antagonists with DBV on Formalin-Induced Nociceptive Responses

**Figure 5:**
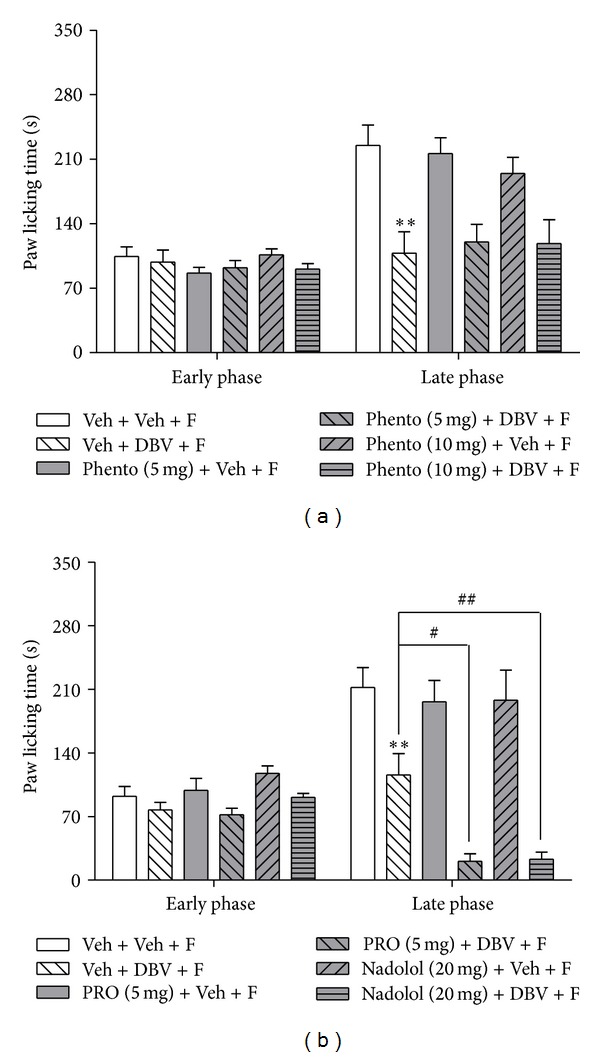
Graphs illustrating the antinociceptive effect of DBV administered together with adrenoceptor antagonists on formalin-induced pain responses during the early and late phases of the formalin test. (a) Injection of DBV (Veh + DBV + F) decreased pain responses compared to the vehicle-injected group (Veh + Veh + F, **P < 0.01). Coadministration of phentolamine with DBV or vehicle [Phento (5, 10 mg) + DBV + F, Phento (5, 10 mg) + Veh + F] did not change formalin-induced pain behaviors as compared to each control group during either the early or late phase. (b) The group receiving vehicle plus DBV and formalin (Veh + DBV + F) showed suppressed pain responses compared to the vehicle- plus-vehicle and formalin-injected group (Veh + Veh + F, **P < 0.01). Injection of propranolol and nadolol with vehicle [PRO (5 mg) + Veh + F, Nadolol (20 mg) + Veh + F] did not change formalin-induced pain responses as compared to the DBV-treated group during the early or late phase. Propranolol and nadolol with DBV treatment [PRO (5 mg) + DBV + F, Nadolol (20 mg) + DBV + F] significantly suppressed formalin-induced pain during the late phase (^#^ *P* < 0.05 and ^##^ *P* < 0.01 as compared with DBV-treated group) but not during the early phase.

The group of mice receiving vehicle plus DBV treatment (Veh + DBV + F, *n* = 7) significantly suppressed pain behaviors in comparison to vehicle-vehicle-injected group (Veh + Veh + F, *n* = 7, **P < 0.01, Figure 5(a)). Systemic injection of the *α*-adrenoceptor antagonist, phentolamine, plus DBV [Phento (5, 10 mg) + DBV + F, *n* = 7, resp.] had no significant effect on formalin-induced nociceptive behavior as compared to the control group during either the early or late phase of the formalin test at any of the doses tested. Similarly coadministration of phentolamine with vehicle [Phento (5, 10 mg) + Veh + F, *n* = 7, resp.] did not affect the paw licking time in either phase (Figure 5(a)). As illustrated in Figure 5(b), treatment of DBV (Veh + DBV + F, *n* = 7) significantly decreased formalin-evoked paw licking time compared to that of vehicle-injected group (Veh + Veh + F, *n* = 7, **P < 0.01), and administration of a *β*-adrenergic receptor antagonist, propranolol with vehicle [PRO (5 mg) + Veh + F, *n* = 7], had no effect on this formalin-induced pain behavior during either the early or late phase. On the other hand, coadministration of propranolol with DBV [PRO (5 mg) + DBV + F, *n* = 7] significantly decreased the paw licking time as compared to the DBV-treated group during the late phase, but not during the early phase of the formalin test (^#^ *P* < 0.05). Additionally, administration of another *β*-adrenergic receptor antagonist, nadolol (which cannot cross the blood-brain barrier), with vehicle [Nadolol (20 mg) + Veh + F, *n* = 7], did not change formalin-induced pain behaviors as compared to vehicle-treated group in either the early or late phase of the formalin test. Similar to propranolol, injection of nadolol in combination with DBV treatment [Nadolol (20 mg) + DBV + F, *n* = 7] produced a significant antinociceptive effect as compared to the vehicle-DBV-treated group during the late phase of the formalin test (^##^ *P* < 0.01). 

**Figure 6:**
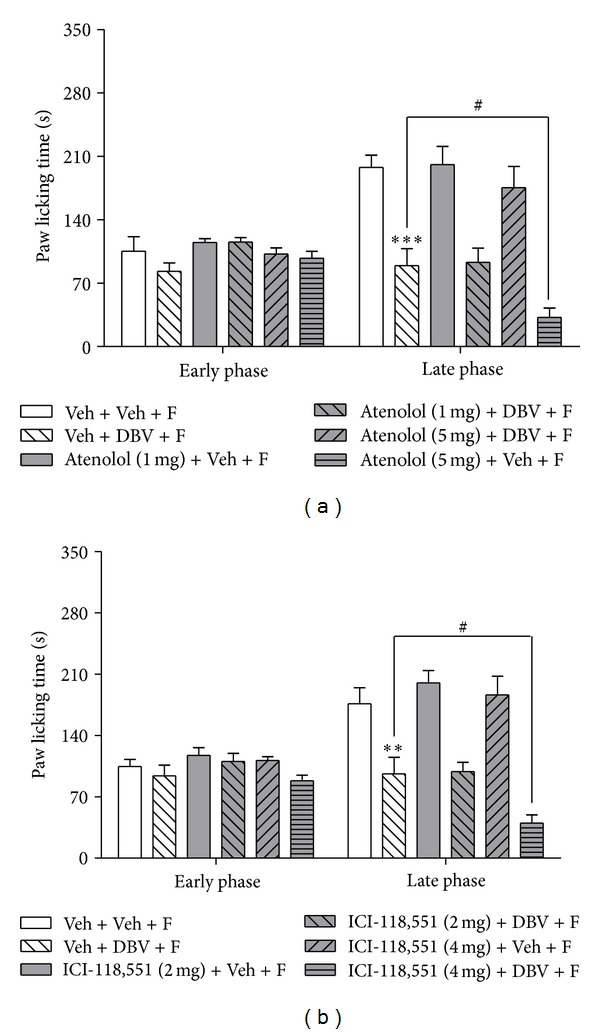
Graphs summarizing the effect of administration of *β*-adrenoceptor subtype antagonists on the antinociceptive effect of DBV on formalin-induced nociceptive behaviors. (a) DBV treatment (Veh + DBV + F) significantly decreased pain behaviors compared with vehicle group (Veh + Veh + F, ***P < 0.001). Coadministration of atenolol with vehicle [Atenolol (1, 5 mg) + Veh + F] did not change pain behaviors as compared to the vehicle-injected group during either the early or late phase. High dose of atenolol pretreatment with DBV [Atenolol (5 mg) + DBV + F] significantly decreased the formalin-induced pain responses as compared to DBV-treated group at the late phase (^#^ *P* < 0.05). (b) Similarly, DBV (Veh + DBV + F) significantly decreased pain behaviors compared to the vehicle group (Veh + Veh + F, **P < 0.01). Intraperitoneal injection of ICI-118,551 with vehicle [ICI-118,551 (2, 4 mg) + Veh + F] had no effect on pain behaviors as compared to the control group during either the early or late phase, whereas administration of a high dose of ICI-118,551 in combination with DBV [ICI-118,551 (4 mg) + DBV + F] significantly decreased the pain responses as compared to the DBV-treated group during the late phase (^#^ *P* < 0.05) but not during the early phase of the formalin test.

We next coadministered either the *β*1-adrenergic receptor antagonist, atenolol (1, 5 mg), or the *β*2-adrenergic receptor antagonist, ICI-118,551 (2, 4 mg), with DBV, to determine the potential contribution of each *β*-adrenoceptor subtype to the antinociceptive effect of DBV on formalin-induced nociceptive behaviors. As shown in Figure 6, administration of DBV (Veh + DBV + F, *n* = 7) significantly decreased pain behaviors compared to that of vehicle-injected group (Veh + Veh + F, *n* = 7, **P < 0.01 and ***P < 0.001), and intraperitoneal administration of either atenolol and ICI-118,551 with vehicle [Atenolol (1, 5 mg) + Veh + F, ICI-118,551 (2, 4 mg) + Veh + F, *n* = 7, resp.] did not change formalin-induced pain behaviors as compared to the vehicle-vehicle-injected group during either the early or late phase of the formalin test. Administration of a low dose of atenolol or ICI-118,551 in combination with administration of DBV [Atenolol (1 mg) + DBV + F, ICI-118,551 (2 mg) + DBV + F, *n* = 7, resp.] had no effect on formalin-induced nociceptive responses as compared to the vehicle-DBV-treated group, whereas administration of a high dose of atenolol or ICI-118,551 with DBV [Atenolol (5 mg) + DBV + F, ICI-118,551 (4 mg) + DBV + F, *n* = 7, resp.] significantly decreased the paw licking time as compared to the vehicle-DBV-treated group during the late phase but not during the early phase of the formalin test (^#^ *P* < 0.05, resp.).

## 4. Discussion

DBV injection into an acupoint has been reported to produce a powerful antinociceptive effect in a variety of animal models of acute and chronic pain. Several possible mechanisms have been proposed to explain the biological processes underlying this DBV-induced antinociceptive effect. For example, it has been shown that DBV injection can activate descending bulbospinal noradrenergic pathways, which in turn activate spinal *α*2-adrenoceptors [2, 11, 22]. On the other hand, it has also been demonstrated that DBV administration can also reduce peripheral leukocyte migration and inflammation-associated increases in TNF-*α* in a mouse air pouch model of inflammation. Thus, DBV injection can produce both antinociceptive and anti-inflammatory effects. It has also been shown that adrenalectomy significantly reduces this DBV-induced suppression of leucocyte migration and TNF-*α* expression [15]. In this regard, DBV stimulation causes increased release of acetylcholine in the spinal cord. This increased acetylcholine acts on spinal muscarinic type 2 receptors leading to a disinhibition mechanism that results in the activation of SPNs that project to the adrenal medulla, and this activation leads to the release of adrenal epinephrine [23]. While this DBV-induced increase of epinephrine from adrenal medulla is one of the mechanisms involved in DBV's anti-inflammatory effects, it is not clear whether a similar mechanism might be involved in DBV-mediated antinociceptive effects.

In the present study, we demonstrated that injection of a high dose of DBV significantly reduces pain behaviors during the late phase of the formalin test, and this DBV-induced antinociceptive effect can be dramatically enhanced by adrenalectomy. On the other hand, adrenalectomy itself had no influence on formalin-induced pain responses. Recently, several investigators have reported that adrenalectomy increases the antinociceptive effect of several analgesics and decreases the hyperalgesia resulting from vagotomy. In this regard, elimination of HPA axis function through adrenalectomy potentiates the antinociceptive effect of the calcium channel blocker, nifedipine, and attenuates its analgesic tolerance [24]. It has been reported that adrenalectomy potentiates morphine sensitivity at the level of the spinal cord and also potentiates the synergistic interaction of supraspinal and spinal morphine action [25]. Moreover, the adrenal medulla is also implicated in vagotomy's pain facilitatory mechanisms, and this hyperalgesic action is reversed by denervation of the adrenal medulla [26]. Khasar et al. have also shown that the hyperalgesic action of the vagal nerve is decreased by suppression of adrenal medulla-derived epinephrine [19]. These findings suggest that an increased release of epinephrine from the adrenal medulla has an important role in peripheral nociception. Based on these reports, it is likely that the DBV-induced antinociceptive effect can be negatively modulated by increases in epinephrine release from adrenal gland. 

Chemical sympathectomy has been reported to enhance the pain-attenuating effect produced by the alpha(2)-adrenoceptor agonist, MPV-2426 [27]. In this regard, we have investigated whether peripheral chemical sympathectomy has an effect on DBV-induced antinociceptive effects. In order to examine the role of sympathetic postganglionic neurons on DBV-induced antinociception, a chemical sympathectomy using intraperitoneal injection of 6-OHDA was performed. Because 6-OHDA cannot cross the blood-brain barrier, it is only able to denervate sympathetic postganglionic neurons and blocks the action of peripheral sympathetic nerve-derived catecholamine [28]. Interestingly, pretreatment with 6-OHDA did not alter DBV-induced antinociception in either phase of the formalin test. Moreover, 6-OHDA by itself did not affect formalin-induced pain responses. These results are similar to those reported in previous studies, which demonstrated that sympathectomy had no effect on baseline pain response and that the sympathetic nervous system has very little effect on pain sensation in healthy subjects [29]. Therefore, our results indicate that the DBV-induced antinociceptive effect is closely associated with adrenal gland-derived epinephrine but not with sympathetic postganglionic neuron derived catecholamines.

In order to evaluate whether increases in peripheral epinephrine can reverse the potentiation of DBV-induced antinociception in adrenalectomized mice, epinephrine was intraperitoneally injected in adrenalectomized mice. The injection of exogenous epinephrine increased peripheral nociception in late phase and restored the DBV-induced increase in antinociception in adrenalectomized mice. These results demonstrated that epinephrine injection is closely involved in peripheral sensitization in late phase and reversed the adrenalectomy-induced facilitation of DBV's antinociceptive effect in comparison to DBV's effect observed in normal mice. It is well established that increases in peripheral epinephrine can affect peripheral nociception. Khasar et al. determined that mechanical hypersensitivity induced by intraplantar injection of epinephrine in the rat was reduced by propranolol suggesting that peripheral *β*-adrenoceptors mediate epinephrine-induced hypersensitivity, and this *β*-adrenoceptor-mediated hyperalgesia is associated with both PKC and PKA second-messenger systems [18, 20]. Since it is also well established that the adrenal medulla is the major source of peripheral epinephrine release in rodents, adrenalectomy-induced facilitation of DBV's antinociceptive effect is likely associated with a decrease in adrenal medulla-derived epinephrine. This also implies that DBV's antinociceptive effect could be partially reduced by a DBV-induced increase in peripheral epinephrine.

Subsequently, we examined whether the suppression of adrenal medulla-derived epinephrine using a PNMT enzyme inhibitor or a peripheral epinephrine receptor antagonist could also potentiate the DBV-induced antinociceptive effect. Epinephrine is synthesized by *N*-methylation of norepinephrine, a reaction catalyzed by PNMT, using a cosubstrate and methyl donor, S-adenosylmethionine (Ado-Met). As a regulator of epinephrine production, PNMT serves as a marker for adrenergic function [30]. Giordano et al. determined that blockade of epinephrine synthesis with intraperitoneal injection of the PNMT enzyme inhibitor, DCMB, resulted in a reduction of buspirone-elevated plasma epinephrine levels [31]. These findings imply that inhibition of the PNMT enzyme is able to reduce the production of endogenous epinephrine, which leads to hypersensitivity to nociceptive stimuli. The results of the present study showed that injection of DCMB alone does not produce an effect on either the early or late phase pain responses in the formalin test, whereas DCMB (10 mg/kg) coadministered with DBV significantly suppressed the formalin-induced pain responses during the late phase. Our data together with that of previous studies demonstrate that DBV-induced antinociception has an influence on the activity of adrenal gland PNMT, which ultimately contributes to a reduction in DBV's antinociceptive effect in the formalin test.

Finally, we examined which subtype of adrenergic receptor is involved in the negative modulation of this DBV-induced antinociceptive effect. It is well established that peripheral epinephrine serves as an endogenous ligand, which can act on both *α*- and *β*-adrenoceptors subtypes. The activation of these receptors via an increase in peripheral epinephrine can lead to peripheral hypersensitivity to noxious stimulus [32, 33]. In the present study, pretreatment with the *α*-adrenoceptor antagonist, phentolamine, was used to examine the role of *α*-adrenoceptors in DBV-induced antinociception. Since phentolamine pretreatment did not affect DBV-induced antinociception, it is reasonable to assume that *α*-adrenoceptors are not associated with DBV-induced antinociception. On the other hand, propranolol, a nonselective *β*-adrenergic antagonist, significantly potentiated DBV-induced antinociception. In order to examine the role of pure peripheral epinephrine, we also used another *β*-adrenergic antagonist, nadolol, which does not cross the blood-brain barrier. These results were similar to those in propranolol-treated mice or adrenalectomized mice, indicating that facilitation of DBV-induced antinociception was mediated by inhibition of peripheral *β*-adrenoceptors, not central nervous system associated *β*-adrenoceptors. Furthermore, we showed that coadministration of either the *β*1-selective adrenergic receptor antagonist, atenolol, or the *β*2-selective adrenergic receptor antagonist, ICI-118,551 with DBV, potentiated the antinociceptive effect of DBV alone. Collectively, these findings demonstrate that DBV treatment results in the release of adrenal medulla-derived epinephrine, which leads to increased peripheral sensitization via activation of *β*1- and *β*2-adrenoceptors, and ultimately leads to partial inhibition of the DBV-induced antinociceptive effect in the mouse formalin test. Thus, injection of DBV into an acupoint has an initial antinociceptive effect on the late phase of the formalin test but at the same time affects the adrenal medulla to increase epinephrine secretion leading to a paradoxical reduction in DBV's antinociceptive effect. 

## 5. Conclusion

The present study shows that adrenalectomy significantly potentiates DBV-induced antinociception, while chemical sympathectomy has no effect. In addition, administration of exogenous epinephrine reverses the enhanced antinociception produced by DBV in adrenalectomized animals. Pretreatment with *α*-adrenergic antagonists did not alter DBV-induced antinociception, whereas *β*-adrenergic antagonists significantly increase DBV-induced antinociception. Collectively, these results demonstrate that suppression of adrenal medulla-derived epinephrine, which acts on *β*-adrenoceptors, can potentiate DBV-induced antinociceptive in the mouse formalin test, suggesting that DBV acupoint stimulation performed in combination with administration of peripheral *β*-adrenoceptor antagonists would be a potential novel strategy for the pain management.
